# Time from Diagnosis and Correlates of Health-Related Quality of Life among Young Adult Colorectal Cancer Survivors

**DOI:** 10.3390/cancers13164045

**Published:** 2021-08-11

**Authors:** Kimberly A. Miller, Julia Stal, Phuong Gallagher, Zhen Weng, David R. Freyer, Jonathan N. Kaslander, Priscilla Marin, Heinz-Josef Lenz, Joel E. Milam, Lauren Govaerts, Afsaneh Barzi

**Affiliations:** 1 Department of Preventive Medicine, Keck School of Medicine of the University of Southern California, Los Angeles, CA 90032, USA; jstal@usc.edu (J.S.); zhenweng@usc.edu (Z.W.); dfreyer@chla.usc.edu (D.R.F.); kaslande@usc.edu (J.N.K.); milamj@hs.uci.edu (J.E.M.); govaerts@usc.edu (L.G.); 2 Department of Dermatology, Keck School of Medicine of the University of Southern California, Los Angeles, CA 90089, USA; 3 The Colon Club, USA; phuong@colonclub.org; 4 Cancer and Blood Disease Institute, Children’s Hospital Los Angeles, Los Angeles, CA 90027, USA; 5 Department of Pediatrics, Keck School of Medicine of the University of Southern California, Los Angeles, CA 90027, USA; 6 USC Norris Comprehensive Cancer Center, Los Angeles, CA 90033, USA; marinp@usc.edu (P.M.); lenz@usc.edu (H.-J.L.); 7 Division of Oncology, Department of Medicine, University of Southern California, Los Angeles, CA 90033, USA; 8 Department of Medical Oncology and Therapeutics Research, City of Hope National Medical Center, Duarte, CA 91010, USA; afsaneh.barzi@myaccesshope.org

**Keywords:** adolescent and young adults, colorectal cancer, survivorship, health-related quality of life

## Abstract

**Simple Summary:**

Young adult survivors of colorectal cancer often undergo intensive and multimodal cancer therapy and may experience impairments in health-related quality of life. However, knowledge regarding the impacts of colorectal cancer on the quality of life of young adults is limited. This study aimed to characterize overall health-related quality of life among young adult colorectal cancer survivors within 36 months of their diagnosis or relapse, to compare those who are shorter versus longer time from initial diagnosis or relapse, and to examine correlates across the distinct domains of quality of life. Such information can inform the development of life stage-appropriate counseling and interventions to maintain and improve health-related quality of life following a colorectal cancer diagnosis for this at-risk patient population.

**Abstract:**

The incidence of colorectal cancer (CRC) is rising among young adults. Health-related quality of life (HRQoL) in survivorship is not well-described in this population. We assessed HRQoL among young adult CRC survivors diagnosed from age 18–39 (AYAs) to examine differences by time from diagnosis, and to identify key correlates. A cross-sectional online survey was administered in collaboration with a national patient advocacy organization. The Functional Assessment of Cancer Therapy (FACT-C) was used to measure HRQoL, which assesses HRQoL globally and across 4 domains: emotional, physical, social, and functional. *T*-tests were conducted to compare HRQoL between survivors who were 6–18 months versus 19–36 months from diagnosis or relapse and multiple linear regression was conducted to identify correlates. The sample (*n* = 196) had a mean age of 32.2(SD ± 4.5); 116 (59.9%) were male; and the self-reported tumor location was colon (39.3%) or rectal (60.7%). The majority (56.4%) were diagnosed with stage 2 disease; 96.9% were non-metastatic. The mean global HRQoL score was 67.7 out of a possible score of 136. Across domains, mean scores were low. Emotional and physical well-being were significantly higher among survivors who were 19–36 months from diagnosis/relapse compared to those 6–18 months from diagnosis/relapse. Longer time from diagnosis and older current age were associated with higher HRQoL, while more intensive treatment and higher clinical disease stage were negatively associated, particularly in the emotional and physical domains. Overall, HRQoL was low in this population, and further research is needed to inform age-appropriate interventions to improve HRQoL for AYA CRC survivors.

## 1. Introduction

Over the past two decades, the significant medical and psychosocial challenges facing adolescent and young adults (AYAs; diagnosed at ages 15–39) with cancer have received considerable attention [1]. In addition to experiencing survival disparities across certain cancer types (e.g., breast cancer, leukemia, osteosarcoma, and rhabdomyosarcoma) [2,3,4,5], AYAs suffer from delays in diagnosis, limited access to appropriate treatment, lower adherence to therapy, low clinical trial enrollment, greater treatment-related toxicity, and greater psychosocial challenges [6,7].

Colorectal cancer (CRC) is the third most common cancer and the second most common cause of cancer mortality in the United States [8]. While incidence rates of CRC in the last decade have dropped by 1.4% per year among individuals aged 50 to 64 years and by 4.0% per year among those aged 65 years and older, rates have risen in adults aged 50 years and older by 1.6% annually, increasing by 22% overall during this time period [9]. Compared to those born in 1950, individuals born circa 1990 (now AYAs, ~age 31) have double the risk of colon cancer and quadruple the risk of rectal cancer [10]. The reasons for these increasing rates are unclear, but may be associated with obesity and lifestyle factors, such as sedentary behavior and poor diet [10]. Based on the increased incidence of CRC among young adults, the U.S. Preventive Services Task Force (USPSTF) issued a revision of screening guidelines in 2021 to lower the starting age of colonoscopy from 50 to 45 years old [11].

Treatment of CRC is multimodal, and surgery, chemotherapy, and radiation are commonly used to manage the disease [12]. The intensive treatment pattern of CRC, as well as symptomology such as fatigue, difficulty sleeping, and/or psychological challenges can substantially impact health-related quality of life (HRQOL), a multidimensional and dynamic concept comprising emotional, physical, social, and functional well-being [13,14]. Among CRC patients, multiple factors impact HRQoL [15]. In a longitudinal study among CRC survivors, HRQoL either marginally improved or remained stable one to three years post-diagnosis but significantly worsened three to ten years post-diagnosis, suggesting long-term changes in HRQoL may become apparent as time from diagnosis increases [16]. By contrast, other research has found that HRQoL among CRC survivors six years post-diagnosis was high [17]. Poor physical health and time from diagnosis may contribute to overall changes in survivor HRQoL, and socio-demographics play a role, such as racial/ethnic minorities, those with lower socioeconomic status, females and those experiencing financial challenges, which have all been associated with lower HRQoL among CRC survivors [18,19].

Much of the existing literature focuses exclusively on older adults and information is lacking regarding the unique HRQoL experiences of AYA CRC survivors [20]. AYA cancer survivors face distinct survivorship challenges due to their age and developmental life stage. Concerns that impact HRQoL that are frequently reported among AYA survivors include financial burden, fertility and reproductive health challenges, issues related to identity development and autonomy, and health information needs [21]. AYAs have been found to have impaired HRQoL compared to non-cancer-affected peers [22]. Thus, the unique needs of AYAs warrants individual research on AYA-specific issues, which has not been comprehensively examined in this population. Filling this crucial gap in HRQoL research is critical to understanding survivorship issues among AYAs with CRC, as this cancer type is becoming increasingly prevalent among young adults [8].

The aim of the current study was to characterize HRQoL among AYA CRC survivors diagnosed between the ages of 18–39 and who were in early survivorship (6–36 months from their diagnosis or relapsed disease). We compared HRQoL by time from diagnosis, examining differences between those 6–18 months versus 19–36 months from diagnosis or relapse to assess whether HRQoL differed in these groups. We then examined potential socio-demographic and clinical correlates of HRQoL both globally and across emotional, physical, social, and functional domains.

## 2. Materials and Methods

A cross-sectional online survey was administered in collaboration with a national patient advocacy organization for young CRC survivors (The Colon Club) between 31 August and 3 September 2020. The Colon Club is based in the United States and its mission is to raise awareness, educate, and help those with CRC, particularly those diagnosed under the age of 45 years. The study was advertised on the group’s Facebook page, and upon survey completion, participants received a $20 electronic gift card. Eligible participants were colon or rectal cancer survivors aged from 18–39 at diagnosis, between 6–36 months from initial diagnosis or the most recent relapse and based in the U.S. All study procedures were approved by the University of Southern California Institutional Review Board (IRB).

As described here and elsewhere [23], steps were taken to ensure the validity and integrity of data and to identify and remove fraudulent responses considering known issues related to social-media recruitment [24]. Participants were first asked a series of questions to determine eligibility using an online Research Electronic Data Capture (REDCap) survey. If participants met eligibility criteria, they were then provided with a study Information Sheet and asked to respond “yes” to consent to study participation. This step of eligibility screening and consent eliminated the threat of automated software or “bots” entering the survey. Additional steps to reduce fraudulent responses included prohibition of duplicate emails, removal of respondents who took the survey in 5 min or less (given the average time of approximately 18 min for survey completion), removal of respondents reporting “improbable” medical treatment responses as reviewed by a medical oncologist (A.B.), and due to a number of respondents reporting improbably high emergency utilization use, removal of those reporting a number of emergency room visits that were two standard deviations from the mean number of visits as outliers.

### 2.1. Measures

Socio-demographic and clinical characteristics: CRC survivors self-reported gender, race/ethnicity, age, stage at diagnosis, whether they had experienced relapsed disease and the year of most recent relapse. For analysis, survivors were coded as having “chronic” disease if they reported relapse or were stage 4 (versus relapse-free and non-metastatic).

Health-related quality of life (HRQoL): Health-related quality of life was measured with the Functional Assessment of Cancer Therapy-Colorectal (FACT-C), a 36-item survey specific to CRC that assesses global HRQoL, as well as the following subdomains: physical well-being, social well-being, emotional well-being, functional well-being, and a subscale specific to colorectal cancer. Questions have a past 7-day recall period and responses are on a 5-point Likert-type scale with higher scores indicating higher HRQoL [25].

Time from diagnosis: CRC survivors were categorized into two groups by the time in months from their initial diagnosis or most recent relapse. Those considered in “short-term” survivorship were 6–18 months from diagnosis/relapse, while those considered in “longer-term” survivorship were 19–36 months. The rationale for distinction of these time periods was that survivors within the first 18 months were likely to either be on or completing therapy and in more active surveillance, whereas those 2–3 years from diagnosis or relapse had likely completed curative therapy and/or were in a chronic disease management state.

Treatment intensity: Treatment intensity was calculated as the sum of self-reported therapies received. Patients were asked to indicate if they received surgery, chemotherapy, radiation, and/or immunotherapy/targeted therapy, with names of specific therapeutic agents listed as examples. The summary score ranged from 0 (no therapy received) to 4 (all four modalities).

Financial toxicity: Financial toxicity was measured with The Comprehensive Score for Financial Toxicity-Functional Assessment of Chronic Illness Therapy (COST-FACIT) questionnaire, an 11-item measure that assesses the degree of financial distress of patients [26,27]. Responses are Likert-type scales with scores ranging from 0–44, with higher scores indicating greater financial distress.

Negative impact of COVID-19: Patients were asked the extent to which COVID-19 disrupted their daily lives using a measure from the Pandemic Stress Index [28]. Responses are on a 5-level Likert-type scale with responses ranging from “not at all” to “extremely” with higher scores reflecting greater daily impacts of the pandemic.

### 2.2. Statistical Analysis

Descriptive statistics, including means and frequencies, were used to describe the sample. Student’s *t*-tests were conducted to examine differences in HRQoL between survivors who were 6–18 months versus 19–36 months from diagnosis or relapse. To examine correlates of HRQoL, socio-demographic and clinical variables were selected for their hypothesized association with HRQoL. Linear regression was conducted with global quality of life and the four subscales (emotional, physical, social, and functional well-being) as outcomes. Due to a majority of non-Latino white participants the survivors were grouped into white versus non-white to examine potential differences by ethnic and racial minoritized status, and as only one-third of survivors had ostomy, this was not controlled for to avoid data loss due to listwise deletion. Significance level was set at *p* < 0.05 and the Benjamini–Hochberg correction was applied to control for the study-wise false discovery rate (Type 1 error) due to multiple comparisons [29]. Statistical analysis was performed using SAS (Version 9.4) (SAS Institute; Cary, NC, USA).

## 3. Results

**Figure 1:**
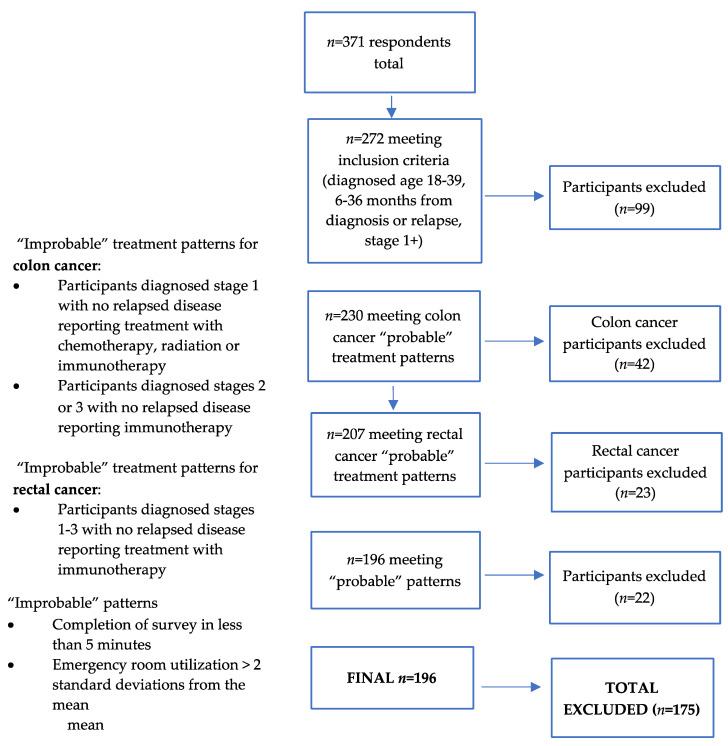
Recruitment and inclusion flow chart.

The sample comprised 196 participants who met all criteria (Figure 1).

Demographic characteristics are displayed in Table 1. Overall mean current age was 32.2 (SD ± 4.5, range 20–42); 116 (59.9%) were male; and tumor location was colon or rectal in 75 (39.3%) and 116 (60.7%), respectively. The majority (110; 60.4%) were diagnosed with stage 2 disease; 96.9% were non-metastatic; 57.7% experienced relapse; and 29.7% had an ostomy.

The majority of survivors were 6–18 months (*n* = 123; 62.6%) from initial diagnosis or relapse, and key demographics including gender, diagnostic stage, cancer type, intensity of treatment, and race/ethnicity did not significantly differ between the two groups. A significantly greater percentage of those diagnosed more recently experienced relapse compared to those further from diagnosis (66.4% versus 43.7%; *p* = 0.002).

Based on recent SEER data, the mean age of diagnosis for CRC among AYAs is 33.6 (SD, 4.8 years), comparable to the age of the study sample [30]. However, the sample differed both in stage distribution and gender compared to SEER data. For SEER 2009–2018 for those aged 15–39, stage distribution for both sexes comprised 37% localized disease, 35% regional disease, and 24% distant disease (4.6% unstaged) [30]. The present study sample had a greater number of survivors diagnosed with regional (particularly stage 2) disease, and a lower number diagnosed with distant (stage 4) disease. Additionally, 60% of survivors in the current sample were male compared to approximately 52% of incident cases in 2010–2015 SEER data [31].The study sample, therefore, reflected survivors with earlier stage disease and a greater proportion of males compared to SEER data. While the stage distribution difference indicates potential survival bias, recent SEER data has shown increases in stage 2 disease among AYAs [31]. This trend may have also contributed to the stage distribution in the present study.

### 3.1. HRQoL Scores

The mean global HRQoL score was 67.7 out of a possible score of 136 (Table 2). In the sample overall, the highest domain-specific score was physical well-being, which was 15.2 out of a possible score of 28, while the lowest scores were in emotional and functional well-being, with scores of 11.7 out of 24 and 11.9 out of 28, respectively.

### 3.2. HRQoL Scores Compared by Time from Diagnosis

Emotional well-being was significantly higher among survivors who were 19–36 months from diagnosis/relapse compared to those 6–18 months from diagnosis/relapse (11.13 for 6–18 months versus 12.56 for 19–36 months; *p* = 0.007). Physical well-being was also significantly higher among survivors 19–36 months from diagnosis/relapse compared to those 6–18 months (14.31 for 6–18 months versus 16.56 for 19–36 months; *p* = 0.007). Social and functional well-being did not significantly differ between groups although mean scores were slightly lower for those who were longer from diagnosis.

### 3.3. Correlates of HRQoL

The relationship between socio-demographic and clinical factors and HRQoL is shown in Table 3. For total HRQoL, only greater treatment intensity was significant in bivariate analyses. For the emotional subscale, in the multivariable model, a higher cancer stage was negatively associated and longer time from diagnosis was positively associated with emotional HRQoL. For the physical subscale, in the multivariable model, older current age and longer time from diagnosis were positively associated with physical HRQoL. Higher cancer stage, greater treatment intensity, and greater COVID daily impacts were negatively associated with physical HRQoL. All remained significant after Benjamini–Hochberg correction. For the social subscale, although several variables were significant in bivariate models, none retained significance in the multivariable model, and no socio-demographic or clinical variables were associated for functional HRQoL at the bivariate level.

## 4. Discussion

This cross-sectional study examined both global and distinct HRQoL domains among AYA CRC survivors. We found overall low HRQoL in this sample, both in global and domain-specific HRQoL scores [15]. Compared to short-term survivors, those longer from diagnosis had higher HRQoL scores in emotional and physical well-being, although no differences were found in social and functional well-being by time from diagnosis. In multivariable models, notably none of the socio-demographic or clinical variables were associated with overall HRQoL, attesting to the importance of examining this construct by specific domains. Across subscales, emotional and physical domains showed significant correlates, including positive associations for age at diagnosis and longer time from diagnosis, and negative associations for higher cancer stage, greater treatment intensity, and negative daily impact of the COVID-19 pandemic.

The low overall HRQoL scores among this sample are concerning, as impaired HRQoL has been associated with higher mortality among survivors of CRC, particularly in the domains of physical and functional well-being [32,33]. In comparison to the current study, other studies have reported higher FACT scores among older adult CRC survivors. In one such study, CRC survivors with a mean age of 59.7 years who were 6 months post-colectomy reported higher HRQoL scores than found in the present study across all domains, with a mean global score of 74.18 (versus 67.66 in the current study) [18]. In that study, physical well-being had an overall mean score of 22.62 among older adults (versus 15.17 in the current study) and functional well-being had an overall mean score of 16.90 (versus 11.90 in the current study). Similar, Rauch et al. (2016) found high quality of life scores among rectal cancer survivors with a median age of 63, similar to, or better than non-cancer affected populations. Thus, the low HRQoL FACT scores among AYA-aged CRC survivors in the present study suggests that disease adaptation may be more challenging in younger adults, indicating a need for further research regarding the unique HRQoL-related challenges AYA CRC survivors face.

It is encouraging that both emotional and physical HRQoL scores were higher for survivors longer from diagnosis or relapse, as well as associated with higher HRQoL in adjusted models, suggesting that as AYA CRC survivors adapt to their lives post-diagnosis and treatment; they may recover both cognitively and physically. The emotional health and affective challenges experienced after a cancer diagnosis and treatment may improve as survivors manage disease-related distress, develop resilience, or receive support to help them cope (e.g., mental health services such as psychotherapy, and/or support from family and friends). Additionally, longer-term survivors may feel more hopeful or less worry that curative therapy has been achieved. Nevertheless, these findings should be interpreted within the context of overall low HRQoL scores; while AYA CRC survivors’ HRQoL may begin to recover over time, those longer from diagnosis continued to report comparatively low scores across domains.

Additionally, stage at diagnosis was associated with lower HRQoL for emotional and physical domains. While recent studies have shown that stage II disease is increasing among AYAs with CRC, particularly among younger age patients [31], many continue to be diagnosed at an advanced stage, and supportive care tailored to these patients is important to maintain HRQoL into survivorship, as these survivors may experience both more difficulty coping with the psychological effects and symptomology of advanced stage disease.

While both emotional and physical HRQoL domains showed differences across the two time periods and were correlated with specific clinical and socio-demographic variables, this was not the case for social or functional HRQoL. These null findings are nevertheless important as they indicate that AYA CRC survivors might experience ongoing deficits in these domains, and that factors customarily perceived to have influence on these domains (e.g., cancer stage, treatment intensity) may not be the most relevant or influential for patients. The challenge remains that clinical variables thought to have negative influence on HRQoL may not have the predicted impacts for survivors. Thus, conventional clinical or demographic classifiers may not be the salient factors that impact HRQoL in this population, which attests to the strong need for more comprehensive and patient-centered research to inform clinical decision-making to support HRQoL outcomes.

Limitations of this research include the social media sample, which does not allow for clinical verification of disease status. The majority of the sample participants were early-stage (stage 2) survivors and predominantly non-Latino white race, and thus do not represent AYA survivors with advanced stage CRC or those from racial/ethnic minoritized groups, both of whom may be at higher risk of poor HRQoL. Although we did not find significant differences between survivors in demographic and clinical characteristics by time from diagnosis, our sample overrepresented early-stage survivors and males compared to national SEER data. Survival bias may explain the greater proportion of early-stage survivors participating in the study and affect the validity of results. If so, the results would likely underestimate HRQoL impairment, even though our findings indicated low scores overall. Similarly, our comparison by time from diagnosis was not truly a longitudinal design, and HRQoL scores were not normed against a control group. For these reasons, appropriate caution must be taken in the interpretation of these findings, which are cross-sectional; future prospective, controlled studies are needed to more precisely understand the dynamic changes in HRQoL over time in this population. Finally, data were collected during the SARS-CoV-2 pandemic (late August–September 2020) making it difficult to disentangle potential impacts on HRQoL related to the pandemic. Nevertheless, we were able to control for self-reported daily impacts of COVID-19 in our multivariable model.

Strengths of the study include a large national sample conducted in partnership with a well-known young adult CRC patient advocacy organization, allowing unique insight into an under-researched and at-risk survivor population.

## 5. Conclusions

CRC is rising among AYAs, and the intensive and multimodal treatment that CRC patients receive can impair HRQoL. AYA cancer patients are a unique patient population with distinct emotional, social, and medical/physical challenges. In this sample of 196 CRC survivors 6–36 months from diagnosis or relapse, HRQoL was low overall, with scores lower than those previously reported among older adult CRC survivors. Those longer from diagnosis had higher HRQoL scores in emotional and physical well-being domains, and time from diagnosis was associated with higher emotional and physical HRQoL in adjusted models, suggesting cognitive and physiological adaptation and resilience. Social and functional HRQoL, by contrast, did not differ by time from diagnosis and had few clinical or socio-demographic correlates. These domains may be areas for future focus and investigation. Overall, these findings indicate that further research is needed to improve HRQoL in this at-risk population and to support the development of tailored, disease and age-appropriate patient counseling and interventions.

## Figures and Tables

**Table 1:** Characteristic of the overall sample and by time from diagnosis (*n* = 196).

Variable	Total	6–18 m ^ *n* = 123 (62.6)	19–36 m ^ *n* = 73 (37.4)
Mean age (SD)	32.2 (4.5)	32.2 (4.5)	32.2 (4.5)
Cancer Type			
Colon	75 (39.3)	53 (44.5)	22 (30.9)
Rectal	116 (60.7)	66 (55.4)	49 (69.0)
Gender			
Male	116 (59.9)	74 (60.7)	42 (59.2)
Female	78 (40.1)	48 (39.3)	29 (40.9)
Stage at diagnosis			
Stage 1	43 (22.1)	26 (21.3)	17 (23.6)
Stage 2	110 (56.4)	66 (54.1)	43 (59.7)
Stage 3	36 (18.4)	26 (21.3)	10 (13.9)
Stage 4	6 (3.1)	4 (3.3)	2 (2.8)
Race/Ethnicity			
Hispanic/Latino	20 (10.4)	12 (10.0)	8 (11.1)
Non-Hispanic White	153 (79.3)	96 (80.0)	57 (79.2)
Black/African-American	13 (6.7)	9 (6.7)	4 (5.6)
Asian/Pacific Islander/Other	7 (3.6)	4 (3.3)	3 (4.2)
Region			
Midwest	37 (19.0)	27 (22.1)	10 (13.7)
Northeast	28 (14.4)	19 (15.6)	9 (12.3)
South	70 (35.9)	42 (34.4)	28 (38.4)
West	60 (30.7)	34 (27.9)	26 (35.6)
Relapse *			
Yes	112 (57.7)	81 (66.4)	31 (43.7)
Ostomy			
Yes	57 (29.7)	35 (29.7)	21 (28.8)

Note: Due to individual item non-response, numbers may not add to 100 percent. * *p* < 0.05. ^ Months from diagnosis.

**Table 2:** Means and *t*-tests to compare quality of life by time from diagnosis among young adult colorectal cancer survivors (*n* = 196).

Variable	Total Sample (*n* = 196)		6–18 m/dx (*n* = 123)	19–36 m/dx (*n* = 73)	t	*p*-Value
	Mean (SD)	95% CI	Mean (SD)	Mean (SD)		
FACT-C Global (0–136)	67.66 (13.15)	[65.8, 69.52]	67.02 (12.25)	68.71 (14.53)	−0.86	0.38
Colorectal cancer scale (0–28)	14.05 (2.90)	[13.64, 14.46]	14.10 (3.17)	13.96 (2.42)	0.33	0.73
Physical well-being (0–28)	15.17 (4.71)	[14.5, 15.84]	14.31 (4.30)	16.56 (5.02)	−3.29	0.001
Social well-being (0–28)	14.88 (4.59)	[14.23, 15.53]	15.32 (4.28)	14.15 (5.00)	1.73	0.08
Emotional well-being (0–24)	11.67 (3.61)	[11.16, 12.18]	11.13 (3.52)	12.56 (3.61)	−2.7	0.007
Functional well-being (0–28)	11.90 (5.04)	[11.19, 12.61]	12.16 (5.05)	11.49 (5.03)	0.88	0.37

**Table 3:** Bivariate and multivariable linear regression for health-related quality of life domains among young adult colorectal cancer survivors (*n* = 196).

Variable	Total QoL		Emotional		Physical		Social		Functional	
	B (se)		B (se)		B (se)		B (se)		B (se)	
	Bivariate	Multi	Bivariate	Multi	Bivariate	Multi	Bivariate	Multi	Bivariate	Multi
Current age ^b^	0.18 (0.21)		0.09 (0.06)		0.18 (0.07) *	0.23 (0.07) *	−0.07 (0.09)		−0.05 (0.08)	-
Gender (ref: male)	−1.06 (1.90)		0.23 (0.53)		0.45 (0.68)		−0.75 (0.67)		−0.38 (0.73)	-
Race (ref: White)	−1.55 (2.25)		0.47 (0.62)		0.01 (0.81)		−2.09 (0.77) **	−1.84 (0.78)	0.20 (0.87)	-
Cancer type (ref: rectal)	−1.88 (1.94)		−0.14 (0.54)		−0.90 (0.70)		−0.23 (0.68)		−0.38 (0.75)	-
Stage at diagnosis ^a^	−0.91 (1.29)		−0.94 (0.35) **	−0.79 (0.34) *	−0.79 (0.46) ^+^	−0.96 (0.45) *	0.94 (0.45) *	0.71 (0.45)	0.32 (0.50)	-
Time from diagnosis ^b^	0.01 (0.01)		0.01 (0.01) **	0.01 (0.01) **	0.01 (0.01) **	0.01 (0.01) *	−0.01 (0.01) *	−0.01 (0.01)	−0.01 (0.01)	-
Treatment intensity ^a^	−2.19 (1.09) *		−0.35 (0.30)		−0.83 (0.39) *	−0.89 (0.38) *	−0.43 (0.38)		−0.50 (0.42)	-
Financial toxicity ^b^	1.01 (0.15)		0.33 (0.04)		0.32 (0.06)		−0.03 (0.06)		0.20 (0.06)	-
COVID impact ^b^	−0.44 (1.03)		0.23 (0.28)		−0.86 (0.36) *	−1.04 (0.37) *	0.43 (0.35)		0.49 (0.38)	-

^+^ *p* < 0.10; * *p* < 0.05; ** *p* < 0.01; (Note: All remained significant after Benjamini–Hochberg correction). ^a^ Ordinal, ^b^ Continuous.

## Data Availability

The data presented in this study are available on request as a limited use dataset from the corresponding author. The data are not publicly available due to privacy considerations for participants.
